# (2*E*)-3-(4-Methyl­phen­yl)-1-(pyridin-3-yl)prop-2-en-1-one

**DOI:** 10.1107/S1600536812032746

**Published:** 2012-07-28

**Authors:** Mauricio de Sousa Oliveiria, Wanderson Costa de Souza, Hamilton B. Napolitano, Allen G. Oliver

**Affiliations:** a Department of Chemistry, State University of Goias, Anapolis, Brazil; b Department of Chemistry and Biochemistry, University of Notre Dame, Notre Dame, IN 46556-5670, USA

## Abstract

The title compound, C_15_H_13_NO, has two crystallographically independent mol­ecules in the asymmetric unit which differ principally in the periplanar angle formed by the benzene and pyridine rings [41.41 (3) and 17.92 (5)°]. The mol­ecules exhibit an *E* conformation between the keto group with respect to the olefin double bond.

## Related literature

For background to related compounds, see: Katsori & Hadjipavlou-Litina (2011 ▶). For biological and medicinal applications of chalcones, see: Bandgar *et al.* (2010 ▶); Juvale *et al.* (2012 ▶); Liu *et al.* (2003 ▶); Sivakumar *et al.* (2011 ▶); Trivedi *et al.* (2007 ▶); Viana *et al.* (2003 ▶). For the synthesis of chalcones, see: Patil *et al.* (2009 ▶).
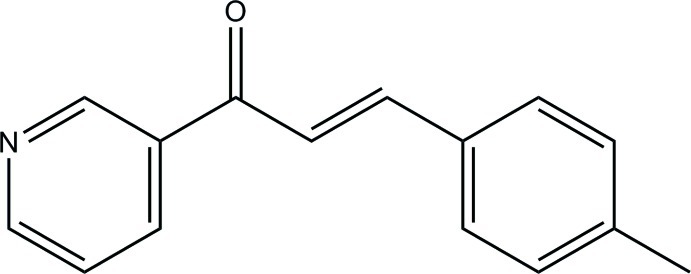


## Experimental

### 

#### Crystal data

C_15_H_13_NO
*M* *_r_* = 223.26
Triclinic, 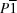

*a* = 5.9026 (7) Å
*b* = 14.2199 (16) Å
*c* = 14.6772 (17) Å
α = 69.654 (2)°
β = 84.231 (2)°
γ = 81.280 (2)°
*V* = 1140.2 (2) Å^3^
*Z* = 4
Mo *K*α radiation
μ = 0.08 mm^−1^
*T* = 120 K
0.19 × 0.08 × 0.05 mm

#### Data collection

Bruker APEXII diffractometer
Absorption correction: numerical (*SADABS*; Bruker, 2012 ▶) *T*_min_ = 0.988, *T*_max_ = 0.997
23125 measured reflections
4692 independent reflections
3684 reflections with *I* > 2σ(*I*)
*R*_int_ = 0.027

#### Refinement

*R*[*F* ^2^ > 2σ(*F* ^2^)] = 0.041
*wR*(*F* ^2^) = 0.105
*S* = 1.03
4692 reflections
309 parameters
H-atom parameters constrained
Δρ_max_ = 0.23 e Å^−3^
Δρ_min_ = −0.23 e Å^−3^

### 

Data collection: *APEX2* (Bruker, 2012 ▶); cell refinement: *SAINT* (Bruker, 2012 ▶); data reduction: *SAINT*; program(s) used to solve structure: *SHELXS97* (Sheldrick, 2008 ▶); program(s) used to refine structure: *SHELXL97* (Sheldrick, 2008 ▶); molecular graphics: *POV-RAY* (Cason, 2003 ▶) and *ORTEP-3* (Farrugia, 1997 ▶); software used to prepare material for publication: *publCIF* (Westrip, 2010 ▶).

## Supplementary Material

Crystal structure: contains datablock(s) I, global. DOI: 10.1107/S1600536812032746/nk2177sup1.cif

Structure factors: contains datablock(s) I. DOI: 10.1107/S1600536812032746/nk2177Isup2.hkl

Supplementary material file. DOI: 10.1107/S1600536812032746/nk2177Isup3.cml

Additional supplementary materials:  crystallographic information; 3D view; checkCIF report

## Figures and Tables

**Fig. 1.**
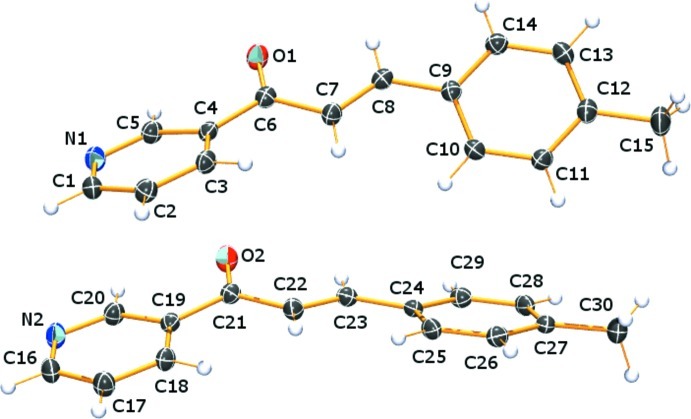
The asymmetric unit of (I). Anisotropic displacement ellipsoids depicted at 50% probabilty. H atoms included as spheres of an arbitrary radius.

**Fig. 2.**
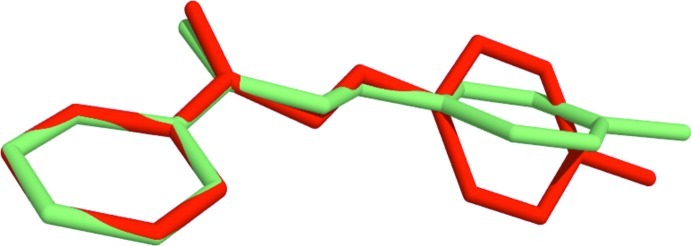
An overlay diagram of the two independent molecules in the asymmetric unit of (I).

## supplementary crystallographic information

### Comment

1,3-Diphenyl-2-propene-1-ones are a class of organic compound that have two aromatic rings bridged by a prop-2-en-1-one group. These compounds belong to the open-chain flavonoid family and possess a wide variety of cytoprotective and modulatory functions, which may have therapeutic potential for multiple diseases. These compounds can be found naturally or can be synthesized by aldol Claisen-Schmidt condensation using alkaline (base) catalysis (Patil *et al.* 2009). Natural chalcones appear mainly as petal pigments and in the heartwood, leaf, fruit and root of different kinds of flora.

A large number of chalcones and their corresponding heterocyclic analogues are a medicinally important class of compounds. It has been shown that chalcones exhibit biological activity against many diseases vectors. Currently, activities of natural and synthetic chalcones include: anticancer (Juvale *et al.* 2012), antioxidant (Sivakumar *et al.* 2011), analgesic (Viana *et al.* 2003), antileishmanial and antimalarial (Liu *et al.* 2003), antimicrobial (Bandgar *et al.* 2010) and antiviral (Trivedi *et al.* 2007) properties.

The pharmacological properties of chalcones are intrinsically linked to the substitution pattern of the two aromatic rings. Their versatility is attributed to the α,β-unsaturated ketene moiety, the conjugated double bonds and the completely delocalized π-electron system on both aromatic rings (Katsori & Hadjipavlou-Litina, 2011).

The structural characterization of (2*E*)-3-(4-methylphenyl)-1-(pyridin-3-yl)prop-2-en-1-one (I) shows that there are two crystallographically independent yet chemically identical molecules in the asymmetric unit (Fig 1). The two molecules differ primarily in the periplanar angle formed by the pyridine and toluene rings: N1—C5(py)···C9—C14(tol) = 41.41 (3)°; N2—C20(py)···C24—C29(tol) = 17.92 (5)°. Differences between the independent molecules are highlighted in the overlay diagram (Fig 2). The different twists within the two molecules is reflected in the torsion angles across the ethylene bond (O1—C6—C7—C8 = 8.6 (2)° and O2—C21—C22—C23 = -14.8 (2)°). Steric interactions between the aromatic and ethylene H atoms are a likely cause for these twists.

### Experimental

(2*E*)-3-(4-Methylphenyl)-1-(pyridin-3-yl)prop-2-en-1-one was obtained using heterogeneous base catalysis. 3-acetyl pyridine 0.270 ml (2.47 mmol) was solubilized in 2 ml of methanol, to which was added 10 ml of 50% potassium hydroxide solution and 0.300 ml (2.47 mmol) of 3-methylbenzaldehyde, successively. The mixture was stirred at ambient conditions and the reaction progress monitored by TLC (Thin Layer Chromatography). Upon reaction completion the mixture was neutralized with a 10% HCl solution. The solid product was washed with water and filtered and subsequently recrystallized from ethanol. The reaction yield was 0.47 g (86%). Suitable crystals of (I) were grown by slow evaporation from a methanol solution..

The Infra-Red spectrum was recorded on a Perkin-Elmer model Spectrum Frontier, from 400 at 4000 cm^-1^ as a KBr pellet: 2916 cm^-1^ CH_3_; 1658 cm^-1^ C═ O; 1596 cm^-1^ C═C (Ethylene); 1011 cm^-1^ C—N (py), 803 cm^-1^ C(Ar). The ^1^H NMR analyses was determined on a Bruker Avance III 500 MHz (11.75 T), spectrometer in DMSO using tetramethyl silane as internal standard. δ 9.23–7.46 (*m*, 8H Ar); 7.46 (*dd*, 2H ethylene (H); 2.48 (*s*, 3H Me).

### Refinement

The hydrogen atoms were initially located from a difference Fourier map and subsequently refined in geometrically calculated positions with methyl C–H distances constrained to 0.98 Å and ethylene and aromatic C–H distances constrained to 0.95 Å. Methyl H atoms were allowed to rotate to minimize the electron density contribution. Thermal parameters of hydrogen atoms were tied to that of the atom to which they are bonded (1.5 × *U*eq for methyl, 1.2 × *U*eq for all others). All non-hydrogen atoms were refined with anisotropic displacement parameters.

### Crystal data

**Table:**

C_15_H_13_NO	*Z* = 4
*M**_r_* = 223.26	*F*(000) = 472
Triclinic, *P*1	*D*_x_ = 1.301 Mg m^−^^3^
Hall symbol: -P 1	Mo *K*α radiation, λ = 0.71073 Å
*a* = 5.9026 (7) Å	Cell parameters from 6635 reflections
*b* = 14.2199 (16) Å	θ = 2.5–26.4°
*c* = 14.6772 (17) Å	µ = 0.08 mm^−^^1^
α = 69.654 (2)°	*T* = 120 K
β = 84.231 (2)°	Block, colourless
γ = 81.280 (2)°	0.19 × 0.08 × 0.05 mm
*V* = 1140.2 (2) Å^3^	

### Data collection

**Table:**

Bruker APEXII diffractometer	4692 independent reflections
Radiation source: fine-focus sealed tube	3684 reflections with *I* > 2σ(*I*)
Bruker TRIUMPH curved-graphite monochromator	*R*_int_ = 0.027
Detector resolution: 8.33 pixels mm^-1^	θ_max_ = 26.5°, θ_min_ = 1.5°
combination of ω and φ–scans	*h* = −7→7
Absorption correction: numerical (*SADABS*; Bruker, 2012	*k* = −17→17
*T*_min_ = 0.988, *T*_max_ = 0.997	*l* = −18→18
23125 measured reflections	

### Refinement

**Table:**

Refinement on *F*^2^	Primary atom site location: structure-invariant direct methods
Least-squares matrix: full	Secondary atom site location: difference Fourier map
*R*[*F*^2^ > 2σ(*F*^2^)] = 0.041	Hydrogen site location: inferred from neighbouring sites
*wR*(*F*^2^) = 0.105	H-atom parameters constrained
*S* = 1.03	*w* = 1/[σ^2^(*F*_o_^2^) + (0.0474*P*)^2^ + 0.4373*P*] where *P* = (*F*_o_^2^ + 2*F*_c_^2^)/3
4692 reflections	(Δ/σ)_max_ = 0.001
309 parameters	Δρ_max_ = 0.23 e Å^−^^3^
0 restraints	Δρ_min_ = −0.23 e Å^−^^3^

### Special details

**Table:**

Geometry. All e.s.d.'s (except the e.s.d. in the dihedral angle between two l.s. planes) are estimated using the full covariance matrix. The cell e.s.d.'s are taken into account individually in the estimation of e.s.d.'s in distances, angles and torsion angles; correlations between e.s.d.'s in cell parameters are only used when they are defined by crystal symmetry. An approximate (isotropic) treatment of cell e.s.d.'s is used for estimating e.s.d.'s involving l.s. planes.
Refinement. Refinement of *F*^2^ against ALL reflections. The weighted *R*-factor *wR* and goodness of fit *S* are based on *F*^2^, conventional *R*-factors *R* are based on *F*, with *F* set to zero for negative *F*^2^. The threshold expression of *F*^2^ > σ(*F*^2^) is used only for calculating *R*-factors(gt) *etc*. and is not relevant to the choice of reflections for refinement. *R*-factors based on *F*^2^ are statistically about twice as large as those based on *F*, and *R*- factors based on ALL data will be even larger.

### Fractional atomic coordinates and isotropic or equivalent isotropic displacement parameters (Å^2^)

**Table:**

	*x*	*y*	*z*	*U*_iso_*/*U*_eq_	
O1	1.21189 (17)	0.89124 (8)	0.50027 (8)	0.0260 (3)	
N1	1.0864 (2)	0.99097 (10)	0.21022 (9)	0.0252 (3)	
C1	0.8702 (3)	1.02931 (11)	0.18543 (11)	0.0231 (3)	
H1	0.8425	1.0611	0.1183	0.028*	
C2	0.6856 (2)	1.02514 (11)	0.25204 (11)	0.0226 (3)	
H2	0.5355	1.0531	0.2307	0.027*	
C3	0.7227 (2)	0.97950 (11)	0.35032 (11)	0.0209 (3)	
H3	0.5986	0.9750	0.3976	0.025*	
C4	0.9451 (2)	0.94052 (10)	0.37837 (10)	0.0185 (3)	
C5	1.1185 (2)	0.94819 (11)	0.30519 (11)	0.0217 (3)	
H5	1.2704	0.9210	0.3244	0.026*	
C6	1.0128 (2)	0.89387 (10)	0.48122 (11)	0.0199 (3)	
C7	0.8380 (2)	0.85094 (11)	0.55766 (10)	0.0207 (3)	
H7	0.6911	0.8455	0.5402	0.025*	
C8	0.8858 (2)	0.81970 (10)	0.65125 (11)	0.0199 (3)	
H8	1.0316	0.8309	0.6647	0.024*	
C9	0.7396 (2)	0.77026 (10)	0.73542 (10)	0.0188 (3)	
C10	0.5392 (2)	0.73304 (11)	0.72701 (11)	0.0211 (3)	
H10	0.4936	0.7400	0.6645	0.025*	
C11	0.4071 (2)	0.68613 (11)	0.80912 (11)	0.0219 (3)	
H11	0.2719	0.6612	0.8019	0.026*	
C12	0.4676 (3)	0.67463 (11)	0.90202 (11)	0.0224 (3)	
C13	0.6673 (3)	0.71086 (11)	0.91037 (11)	0.0238 (3)	
H13	0.7127	0.7034	0.9730	0.029*	
C14	0.8011 (2)	0.75763 (11)	0.82884 (11)	0.0219 (3)	
H14	0.9372	0.7816	0.8365	0.026*	
C15	0.3179 (3)	0.62614 (13)	0.99064 (12)	0.0319 (4)	
H15A	0.2129	0.5881	0.9739	0.048*	
H15B	0.2290	0.6787	1.0131	0.048*	
H15C	0.4146	0.5801	1.0425	0.048*	
O2	1.03753 (17)	0.70034 (8)	0.34296 (8)	0.0276 (3)	
N2	0.9368 (2)	0.84417 (10)	0.05356 (9)	0.0258 (3)	
C16	0.7218 (3)	0.87995 (11)	0.02547 (11)	0.0244 (3)	
H16	0.7003	0.9168	−0.0414	0.029*	
C17	0.5298 (3)	0.86595 (11)	0.08880 (11)	0.0241 (3)	
H17	0.3807	0.8919	0.0654	0.029*	
C18	0.5586 (2)	0.81352 (11)	0.18673 (10)	0.0205 (3)	
H18	0.4295	0.8028	0.2316	0.025*	
C19	0.7788 (2)	0.77691 (10)	0.21845 (10)	0.0179 (3)	
C20	0.9597 (2)	0.79400 (11)	0.14810 (11)	0.0225 (3)	
H20	1.1105	0.7679	0.1693	0.027*	
C21	0.8356 (2)	0.72037 (10)	0.32200 (10)	0.0193 (3)	
C22	0.6481 (2)	0.68910 (11)	0.39596 (10)	0.0207 (3)	
H22	0.4946	0.7185	0.3817	0.025*	
C23	0.6925 (2)	0.61986 (11)	0.48271 (10)	0.0195 (3)	
H23	0.8487	0.5924	0.4928	0.023*	
C24	0.5285 (2)	0.58134 (10)	0.56403 (10)	0.0182 (3)	
C25	0.2975 (2)	0.62264 (11)	0.56456 (10)	0.0200 (3)	
H25	0.2401	0.6759	0.5089	0.024*	
C26	0.1526 (2)	0.58659 (11)	0.64536 (11)	0.0213 (3)	
H26	−0.0032	0.6159	0.6444	0.026*	
C27	0.2294 (3)	0.50814 (11)	0.72827 (10)	0.0208 (3)	
C28	0.4577 (3)	0.46570 (11)	0.72671 (10)	0.0222 (3)	
H28	0.5134	0.4112	0.7818	0.027*	
C29	0.6049 (2)	0.50143 (11)	0.64645 (10)	0.0208 (3)	
H29	0.7600	0.4713	0.6473	0.025*	
C30	0.0694 (3)	0.47278 (12)	0.81652 (11)	0.0287 (4)	
H30A	−0.0431	0.5297	0.8204	0.043*	
H30B	0.1578	0.4463	0.8752	0.043*	
H30C	−0.0106	0.4194	0.8114	0.043*	

### Atomic displacement parameters (Å^2^)

**Table:**

	*U*^11^	*U*^22^	*U*^33^	*U*^12^	*U*^13^	*U*^23^
O1	0.0183 (5)	0.0319 (6)	0.0242 (6)	−0.0048 (4)	−0.0003 (4)	−0.0046 (5)
N1	0.0232 (6)	0.0308 (7)	0.0206 (7)	−0.0072 (5)	0.0043 (5)	−0.0073 (6)
C1	0.0272 (8)	0.0238 (7)	0.0171 (7)	−0.0070 (6)	−0.0004 (6)	−0.0042 (6)
C2	0.0204 (7)	0.0219 (7)	0.0238 (8)	−0.0026 (6)	−0.0011 (6)	−0.0057 (6)
C3	0.0185 (7)	0.0222 (7)	0.0213 (8)	−0.0042 (6)	0.0041 (6)	−0.0072 (6)
C4	0.0202 (7)	0.0161 (7)	0.0192 (7)	−0.0056 (5)	0.0019 (6)	−0.0055 (6)
C5	0.0171 (7)	0.0240 (7)	0.0231 (8)	−0.0036 (6)	0.0011 (6)	−0.0070 (6)
C6	0.0196 (7)	0.0179 (7)	0.0218 (8)	−0.0019 (5)	0.0008 (6)	−0.0067 (6)
C7	0.0191 (7)	0.0218 (7)	0.0202 (8)	−0.0043 (6)	0.0010 (6)	−0.0054 (6)
C8	0.0178 (7)	0.0181 (7)	0.0232 (8)	−0.0026 (5)	−0.0003 (6)	−0.0064 (6)
C9	0.0200 (7)	0.0168 (7)	0.0186 (7)	−0.0011 (5)	−0.0006 (6)	−0.0054 (6)
C10	0.0231 (7)	0.0237 (7)	0.0187 (7)	−0.0043 (6)	−0.0007 (6)	−0.0092 (6)
C11	0.0210 (7)	0.0236 (7)	0.0229 (8)	−0.0068 (6)	0.0023 (6)	−0.0093 (6)
C12	0.0238 (7)	0.0205 (7)	0.0210 (8)	−0.0026 (6)	0.0033 (6)	−0.0058 (6)
C13	0.0267 (8)	0.0270 (8)	0.0173 (8)	−0.0021 (6)	−0.0031 (6)	−0.0071 (6)
C14	0.0200 (7)	0.0227 (7)	0.0232 (8)	−0.0033 (6)	−0.0030 (6)	−0.0074 (6)
C15	0.0324 (9)	0.0383 (9)	0.0219 (9)	−0.0096 (7)	0.0070 (7)	−0.0065 (7)
O2	0.0176 (5)	0.0349 (6)	0.0241 (6)	−0.0022 (4)	−0.0027 (4)	−0.0023 (5)
N2	0.0243 (7)	0.0301 (7)	0.0195 (7)	−0.0064 (5)	0.0032 (5)	−0.0037 (6)
C16	0.0293 (8)	0.0244 (8)	0.0162 (8)	−0.0045 (6)	−0.0005 (6)	−0.0025 (6)
C17	0.0211 (7)	0.0268 (8)	0.0210 (8)	−0.0016 (6)	−0.0031 (6)	−0.0037 (6)
C18	0.0185 (7)	0.0222 (7)	0.0192 (8)	−0.0034 (6)	0.0028 (6)	−0.0058 (6)
C19	0.0194 (7)	0.0161 (7)	0.0183 (7)	−0.0037 (5)	0.0002 (5)	−0.0058 (6)
C20	0.0174 (7)	0.0250 (8)	0.0231 (8)	−0.0038 (6)	0.0003 (6)	−0.0054 (6)
C21	0.0194 (7)	0.0175 (7)	0.0204 (8)	−0.0020 (6)	−0.0005 (6)	−0.0057 (6)
C22	0.0190 (7)	0.0237 (7)	0.0183 (8)	−0.0025 (6)	−0.0006 (6)	−0.0059 (6)
C23	0.0185 (7)	0.0215 (7)	0.0197 (7)	−0.0025 (6)	−0.0014 (6)	−0.0086 (6)
C24	0.0221 (7)	0.0189 (7)	0.0158 (7)	−0.0053 (6)	−0.0020 (5)	−0.0070 (6)
C25	0.0231 (7)	0.0197 (7)	0.0161 (7)	−0.0029 (6)	−0.0032 (6)	−0.0038 (6)
C26	0.0209 (7)	0.0230 (7)	0.0214 (8)	−0.0044 (6)	0.0006 (6)	−0.0091 (6)
C27	0.0278 (8)	0.0206 (7)	0.0166 (7)	−0.0093 (6)	0.0006 (6)	−0.0073 (6)
C28	0.0302 (8)	0.0196 (7)	0.0154 (7)	−0.0057 (6)	−0.0050 (6)	−0.0019 (6)
C29	0.0211 (7)	0.0200 (7)	0.0213 (8)	−0.0020 (6)	−0.0036 (6)	−0.0065 (6)
C30	0.0337 (9)	0.0303 (8)	0.0210 (8)	−0.0112 (7)	0.0042 (7)	−0.0057 (7)

### Geometric parameters (Å, º)

**Table:**

O1—C6	1.2269 (17)	C25—C26	1.381 (2)
N1—C5	1.3332 (19)	C26—C27	1.395 (2)
N1—C1	1.3427 (19)	C27—C28	1.392 (2)
C1—C2	1.383 (2)	C27—C30	1.506 (2)
C2—C3	1.385 (2)	C28—C29	1.383 (2)
C3—C4	1.389 (2)	C1—H1	0.9500
C4—C5	1.395 (2)	C2—H2	0.9500
C4—C6	1.491 (2)	C3—H3	0.9500
C6—C7	1.4758 (19)	C5—H5	0.9500
C7—C8	1.334 (2)	C7—H7	0.9500
C8—C9	1.4596 (19)	C8—H8	0.9500
C9—C14	1.397 (2)	C10—H10	0.9500
C9—C10	1.400 (2)	C11—H11	0.9500
C10—C11	1.384 (2)	C13—H13	0.9500
C11—C12	1.392 (2)	C14—H14	0.9500
C12—C13	1.388 (2)	C15—H15A	0.9800
C12—C15	1.509 (2)	C15—H15B	0.9800
C13—C14	1.383 (2)	C15—H15C	0.9800
O2—C21	1.2270 (17)	C16—H16	0.9500
N2—C20	1.3315 (19)	C17—H17	0.9500
N2—C16	1.342 (2)	C18—H18	0.9500
C16—C17	1.384 (2)	C20—H20	0.9500
C17—C18	1.384 (2)	C22—H22	0.9500
C18—C19	1.387 (2)	C23—H23	0.9500
C19—C20	1.395 (2)	C25—H25	0.9500
C19—C21	1.497 (2)	C26—H26	0.9500
C21—C22	1.4742 (19)	C28—H28	0.9500
C22—C23	1.333 (2)	C29—H29	0.9500
C23—C24	1.4614 (19)	C30—H30A	0.9800
C24—C29	1.399 (2)	C30—H30B	0.9800
C24—C25	1.401 (2)	C30—H30C	0.9800
			
C5—N1—C1	116.27 (13)	C3—C2—H2	120.5
N1—C1—C2	123.74 (14)	C2—C3—H3	120.7
C1—C2—C3	118.99 (14)	C4—C3—H3	120.7
C2—C3—C4	118.63 (13)	N1—C5—H5	117.7
C3—C4—C5	117.69 (13)	C4—C5—H5	117.7
C3—C4—C6	124.68 (13)	C8—C7—H7	119.9
C5—C4—C6	117.60 (12)	C6—C7—H7	119.9
N1—C5—C4	124.67 (13)	C7—C8—H8	116.3
O1—C6—C7	121.61 (13)	C9—C8—H8	116.3
O1—C6—C4	119.53 (13)	C11—C10—H10	119.8
C7—C6—C4	118.86 (12)	C9—C10—H10	119.8
C8—C7—C6	120.17 (13)	C10—C11—H11	119.2
C7—C8—C9	127.43 (13)	C12—C11—H11	119.2
C14—C9—C10	117.80 (13)	C14—C13—H13	119.5
C14—C9—C8	119.54 (13)	C12—C13—H13	119.5
C10—C9—C8	122.65 (13)	C13—C14—H14	119.4
C11—C10—C9	120.46 (14)	C9—C14—H14	119.4
C10—C11—C12	121.58 (14)	C12—C15—H15A	109.5
C13—C12—C11	117.93 (13)	C12—C15—H15B	109.5
C13—C12—C15	121.11 (14)	H15A—C15—H15B	109.5
C11—C12—C15	120.95 (14)	C12—C15—H15C	109.5
C14—C13—C12	121.01 (14)	H15A—C15—H15C	109.5
C13—C14—C9	121.21 (14)	H15B—C15—H15C	109.5
C20—N2—C16	116.36 (13)	N2—C16—H16	118.2
N2—C16—C17	123.51 (14)	C17—C16—H16	118.2
C16—C17—C18	118.90 (14)	C16—C17—H17	120.5
C17—C18—C19	119.07 (13)	C18—C17—H17	120.5
C18—C19—C20	117.21 (13)	C17—C18—H18	120.5
C18—C19—C21	124.84 (13)	C19—C18—H18	120.5
C20—C19—C21	117.95 (12)	N2—C20—H20	117.5
N2—C20—C19	124.94 (13)	C19—C20—H20	117.5
O2—C21—C19	119.07 (13)	C26—C25—H25	119.8
C22—C21—C19	119.23 (12)	C23—C22—H22	119.8
C23—C22—C21	120.45 (13)	C21—C22—H22	119.8
O2—C21—C22	121.69 (13)	C22—C23—H23	116.3
C22—C23—C24	127.46 (13)	C24—C23—H23	116.3
C29—C24—C25	117.97 (13)	C24—C25—H25	119.8
C29—C24—C23	119.10 (13)	C25—C26—H26	119.2
C25—C24—C23	122.92 (13)	C27—C26—H26	119.2
C26—C25—C24	120.50 (13)	C29—C28—H28	119.4
C25—C26—C27	121.60 (14)	C27—C28—H28	119.4
C28—C27—C26	117.77 (13)	C28—C29—H29	119.5
C28—C27—C30	121.84 (13)	C24—C29—H29	119.5
C26—C27—C30	120.38 (14)	C27—C30—H30A	109.5
C29—C28—C27	121.19 (13)	C27—C30—H30B	109.5
C28—C29—C24	120.94 (13)	H30A—C30—H30B	109.5
N1—C1—H1	118.1	C27—C30—H30C	109.5
C2—C1—H1	118.1	H30A—C30—H30C	109.5
C1—C2—H2	120.5	H30B—C30—H30C	109.5
			
C5—N1—C1—C2	−0.9 (2)	C20—N2—C16—C17	−0.8 (2)
N1—C1—C2—C3	0.3 (2)	N2—C16—C17—C18	0.9 (2)
C1—C2—C3—C4	0.7 (2)	C16—C17—C18—C19	0.2 (2)
C2—C3—C4—C5	−1.1 (2)	C17—C18—C19—C20	−1.2 (2)
C2—C3—C4—C6	176.94 (13)	C17—C18—C19—C21	179.11 (14)
C1—N1—C5—C4	0.4 (2)	C16—N2—C20—C19	−0.3 (2)
C3—C4—C5—N1	0.5 (2)	C18—C19—C20—N2	1.3 (2)
C6—C4—C5—N1	−177.64 (13)	C21—C19—C20—N2	−178.99 (14)
C3—C4—C6—O1	−156.74 (14)	C18—C19—C21—O2	−172.83 (14)
C5—C4—C6—O1	21.3 (2)	C20—C19—C21—O2	7.5 (2)
C3—C4—C6—C7	23.9 (2)	C18—C19—C21—C22	7.9 (2)
C5—C4—C6—C7	−158.09 (13)	C20—C19—C21—C22	−171.81 (13)
O1—C6—C7—C8	8.6 (2)	O2—C21—C22—C23	−14.8 (2)
C4—C6—C7—C8	−171.99 (13)	C19—C21—C22—C23	164.46 (13)
C6—C7—C8—C9	−176.07 (13)	C21—C22—C23—C24	179.32 (13)
C7—C8—C9—C14	−169.49 (14)	C22—C23—C24—C29	173.98 (14)
C7—C8—C9—C10	11.4 (2)	C22—C23—C24—C25	−7.7 (2)
C14—C9—C10—C11	0.5 (2)	C29—C24—C25—C26	1.6 (2)
C8—C9—C10—C11	179.57 (13)	C23—C24—C25—C26	−176.72 (13)
C9—C10—C11—C12	0.2 (2)	C24—C25—C26—C27	−0.4 (2)
C10—C11—C12—C13	−0.7 (2)	C25—C26—C27—C28	−1.0 (2)
C10—C11—C12—C15	178.11 (14)	C25—C26—C27—C30	177.93 (14)
C11—C12—C13—C14	0.5 (2)	C26—C27—C28—C29	1.3 (2)
C15—C12—C13—C14	−178.31 (14)	C30—C27—C28—C29	−177.61 (14)
C12—C13—C14—C9	0.2 (2)	C27—C28—C29—C24	−0.2 (2)
C10—C9—C14—C13	−0.7 (2)	C25—C24—C29—C28	−1.3 (2)
C8—C9—C14—C13	−179.79 (13)	C23—C24—C29—C28	177.09 (13)
